# Prevalence of self-reported musculoskeletal disorders of the hand and associated conducted therapy approaches among dentists and dental assistants in Germany

**DOI:** 10.1371/journal.pone.0241564

**Published:** 2020-11-06

**Authors:** Yvonne Haas, Antonia Naser, Jasmin Haenel, Laura Fraeulin, Fabian Holzgreve, Christina Erbe, Werner Betz, Eileen M. Wanke, Doerthe Brueggmann, Albert Nienhaus, David A. Groneberg, Daniela Ohlendorf

**Affiliations:** 1 Institute of Occupational Medicine, Social Medicine and Environmental Medicine, Goethe-University, Frankfurt am Main, Germany; 2 Department of Orthodontics, School of Dentistry, University Medical Centre of the Johannes Gutenberg University Mainz, Mainz, Germany; 3 Institute of Dentistry, Goethe-University, Frankfurt am Main, Germany; 4 Principles of Prevention and Rehabilitation Department (GPR), Institute for Statutory Accident Insurance and Prevention in the Health and Welfare Services (BGW), Hamburg, Germany; Gazi University, Faculty of Health Sciences, TURKEY

## Abstract

**Background:**

Dental professionals are subjected to higher risks for musculoskeletal disorders (MSDs) than other professional groups, especially the hand region. This study aims to investigate the prevalence of hand complaints among dentists (Ds) and dental assistants (DAs) and examines applied therapies.

**Methods:**

For this purpose, an online questionnaire analysed 389 Ds (240female/149male) and 406 DAs (401female/5male) working in Germany. The self-reported data of the two occupational groups were compared with regard to the topics examined. The questionnaire was based on the Nordic Questionnaire (self-reported lifetime, 12-month and 7-day MSDs prevalence of the hand, the conducted therapy and its success), additional occupational and sociodemographic questions as well as questions about specific medical conditions.

**Results:**

30.8% of Ds affirmed MSDs in the hand at any time in their lives, 20.3% in the last twelve months and 9.5% in the last seven days. Among DAs, 42.6% reported a prevalence of MSDs in the hand at any time in their lives, 31.8% in the last 12 months and 15.3% in the last seven days. 37.5% of the Ds and 28.3% of the DAs stated that they had certain treatments. For both, Ds and DAs, physiotherapy was the most frequently chosen form of therapy. 89.7% of Ds and 63.3% of DAs who received therapy reported an improvement of MSDs.

**Conclusion:**

Although the prevalence of MSDs on the hand is higher among DAs than among Ds, the use of therapeutic options and the success of therapy is lower for DAs compared to Ds.

## Introduction

The work of dentists (Ds) and dental assistants (DAs) involves several occupational health risk factors that are higher in comparison to other occupational groups [1]. Such risk factors include instrument vibration, poor lighting conditions, poor positioning of the dental professionals when working on the patients or poor positioning of the patients [2–4]. The impact on the musculoskeletal system in dentistry is high, the upper extremity being especially very vulnerable [4–9]. Lietz et al. [10] reviewed the prevalence of muscular-skeletal disorders (MSDs) among dental professionals in different countries. In addition to neck, shoulder or upper and lower back problems, hand problems also occur [6, 11–17]. In fact, after the shoulders, the hands in the upper extremities are the next most frequently affected body part [10]. The 12-month prevalence of MSDs in the hand varies between 25.0% and 49.7% [4, 9, 11–13, 18–20]. Other international studies report hand complaints ranging from 8.4% to 66.7% [8, 14, 15, 21–24]. Other research showed that 19.2% to 50.0% of dental students develop MSDs on the hand during their studies [25, 26].

The characteristics of the dental instruments (weight, shape, size, structure) can influence the development of pathologies of the hand [27]. In particular, the vibrations emitted by some instruments are risk factors [28]. Apart from the practical work on the patients, dental professionals are required to complete administrative work at the desk. Adopting awkward postures and specific computer movements while operating the computer can lead to MSDs in the regions of the hand [29].

The potential consequences of MSDs can be persistent for the affected person and have a high probability of developing into a chronic condition [1]; this is especially the case for hand complaints which have a higher risk of chronicity [30]. Rafie et al. [6] reported that, as a result of MSDs in general, about 15.0% of Ds have had to leave their hospital positions or reduce their working hours. Longer absences and early retirement may also result from MSDs [3, 12, 31, 32]. Gupta et al. [33] concluded that the occurrence of MSDs (29.5%) is the most frequent cause of the early retirement of Ds. Besides MSDs, there are clinical pictures at the upper extremities that are increasingly appearing among dental staff [27]; these include, for example, arthritis at 5.0% and tendinitis at 8.7%, which are more frequent in comparison to other occupational groups studied (e.g. doctors and lawyers) [34]. In addition, the prevalence of osteoarthritis in the fingers (48.0%) [35] and carpal tunnel syndrome (ranging from 9.0% to 30.5%) [18, 22, 36] have also been reported.

Therapeutic measures to reduce the severity of MSDs include various sports activities (physiotherapy, weight training, stretching, acupuncture or yoga) [13, 16, 37, 38] and conventional therapies (drug-based pain therapy, surgery or alternative therapy concepts) [39]. The American Dental Association (ADA), for example, reports a proportion of approximately 20.0% of Ds with MSDs in the upper extremities who have undergone surgery [40]. Moreover, the tasks or work areas should be changed regularly and breaks should be scheduled to combat the strain on the musculoskeletal system [7, 13, 14]. With regard to relational measures, several ergonomic studies at the dental workplace confirm that an improvement in ergonomics (e.g. with regard to instruments, the practitioner’s as well as the patient’s chair and the arrangement of the working environment) leads to a decrease in MSDs [37, 41–44].

Although the prevalence of MSDs in the hand area is high worldwide and hands undoubtedly play an important role in the work of dental professionals, there are no current studies on the prevalence of MSDs in the hand, particularly in Germany. Therefore, the aim of the present analysis is to collect current numbers on the prevalence of MSDs among dental professionals and dental assistants in Germany and to detect possible similarities or differences. On the other hand, the different therapeutic approaches used by affected Ds and DAs will be presented; it will be analysed as to what extent therapies are actually applied and what type of therapies are used.

## Material and methods

The observational study was designed as a cross-sectional study. The data were collected between May 2018 and May 2019 anonymously with an online questionnaire distributed via the SoSci Survey platform [45].

### Subjects

A total of 2548 persons followed the link to the anonymous online questionnaire, of which, 795 completed the questionnaire and were included for analysis. Of the 795 individuals, 389 were Ds (240female/149male) and 406 were DAs (401female/5male). 79.3% of the DAs were already in employment while 20.7% were in training. The age of the participating Ds was between 19 and 75 years, with a median age of 39.5 years (1st quartile 31.0 years and 3rd quartile 53.0 years), whilst for the DAs, the age ranged between 18 and 68 years, with a median age of 28.0 years (1st quartile 23.0 years and 3rd quartile 38.0 years).

The inclusion criteria were the following: a job or apprentice position in Germany and the membership of a professional DA society or an apprentice DA contract and a minimum age of 18 years. Exclusion criteria were participants who were not classified as DAs according to the official definition of the society (e.g. dental hygienists). Furthermore, dental students were excluded from the evaluation due to their partial lack of practical experience; this is in contrast to DAs in training, who have a permanent position in a dental practice from the very start of their training. In addition, respondents with missing important information (e.g. gender) and incomplete questionnaires were excluded.

All participants worked in the following disciplines: general dentistry, oral surgery/maxillofacial surgery, endodontology, orthodontics, prosthetics, pediatric dentistry and periodontology.

All data collected in the questionnaire is self-reported by the Ds respectively by the DAs. The diagnoses of the medical conditions are based on self-reports as well. We have not collected other medical data.

At the beginning of the online survey, a declaration of informed consent had to be accepted. The study was approved by the local medical ethics committee of the medical faculty (Goethe University Frankfurt; No. 356/17).

### Questionnaire

The questionnaire consisted of questions from three areas. Firstly, the questionnaire contained variables from the Nordic Questionnaire [46, 47]. These included questions on the 7-day prevalence, 12-month prevalence and lifetime prevalence of hand pain. In addition, questions were asked about any therapy conducted and its success. Secondly, socio-demographic questions (gender, age, education in Germany, size, body weight, body height and handedness), job-related questions (professional group, specialisation, professional experience in years, average total working time per week in hours, average treatment time per week in hours, average administrative time per week in hours, change of job, federate state of dental office and practice type) and questions about physical activity based on the questionnaire by Meyer et al. [48] were collected. Thirdly, questions about specific medical conditions of the hands, which are frequently associated with dental professionals, were presented.

The Nordic Questionnaire, according to Kuorinka et al. [47], has been validated and is used worldwide for various studies to investigate self-reported MSDs in different fields of work. The complaints are queried using 28 multiple-choice questions covering the nine body regions of the neck, shoulder, elbow, wrist, thoracic spine, lumbar spine, hip, knee and ankle. The 7-day prevalence and the 12-month prevalence are asked in each case. Furthermore, the Nordic Questionnaire asks about the lifetime prevalence of MSDs, professional and private consequences of MSDs, the duration of the problems, possible accidents and therapies used in the three areas of the neck, shoulder and lower back.

Meyer et al. [48] investigated dentists in Germany in their questionnaire regarding job-related complaints. In addition to stress factors and occupational dermatoses, spinal column strains were examined. Sociodemographic (gender, age, education in Germany, body height, body weight and handedness), occupational (professional group, specialisation, professional experience in years, average total working time per week in hours, average treatment time per week in hours, average administrative time per week in hours, change of job, federate state of dental office and practice type) and other job-related data were collected.

Furthermore, the questionnaire of the present study consisted of questions on specific medical conditions that typically occur in dental professionals at the upper extremities; these include rheumatism, arthrosis, carpal and cubital tunnel syndromes and tendovaginitis [35, 49–54].

For a pretest, the questionnaire was subjected to 13 individuals within the target group. Subsequently, after a re-evaluation, adjustments were made to provide a better understanding and to ensure functionality.

### Recruitment

The participants were approached via different recruitment strategies. The study was distributed via several German social networks used by Ds, DAs and DAs in training. Furthermore, the study was shared with dental professionals and their internal networks with information distributed through the dental chambers nationwide, while flyers were sent to vocational schools of DAs in the state of Hessen. Moreover, the project was made public at the German Dentists' Day in Frankfurt am Main (Germany) on 9.-11.10.2018 and at the International Dental Show (IDS) in Cologne (Germany) on 15.-16.3.2019 (which are both considered as the largest events for Ds and DAs in Germany). The study was also presented via articles in the specialist journal "Die Zahnarzt Woche (DZW)" and the journal "Zahnärztliche Mitteilungen (ZM)".

### Data editing and analysis

After collecting the data via SoSci Survey [45], it was exported to "Microsoft Excel 16.5 [55]" to process the information and to encode open variables. Improvements were made regarding the logic of the answers. Some specialisations were grouped under "others" and, if frequently reported, new groups were created, e.g. prosthetics, paediatric dentistry and periodontology, as well as dental hygiene. Therapies that did not fit into the physiotherapy, medicine and surgery groups were added to the category “others”. Therapy concepts of physiotherapy were also divided into different subcategories. In addition, obvious errors in body weight or height etc., were corrected. Uncorrectable answers were removed from the evaluation.

The data was transferred to IBM Statistics SPSS 26 [56] in order to perform the descriptive analysis. The median and interquartile distances were calculated for all non-normally distributed metric data. The non-normal distribution was evaluated using the Kolmogorov-Smirnov test. Furthermore, to test the data for differences, the Chi-square test was used for the nominal data and the Wilcoxon-Mann-Whitney test for the quantitative data. Significant relationships were marked with an asterisk in the corresponding tables and figures in the "Results" section.

## Results

### Individual information of the study population

Table 1 shows the self-reported demographic data of the respondents, divided into Ds and DAs. Significant differences (p = 0.01) were found for age, body weight, BMI and height. The differences between the DAs and Ds in physical activity and muscle training were significant (p = 0.01).

**Table 1:** Individual data of the study population–Ds and DAs.

	Ds	DAs
**Sex n (%)**		
Female	240 (61.7)	401 (98.8)
Male	149 (38.3)	5 (1.2)
Age (Years)*		
$\tilde{x}$ (I50)	39.5 (22.0)	28 (15.0)
Body height (cm)*		
$\tilde{x}$ (I50)	172 (12.3)	166 (8.0)
Body weight (kg)*		
$\tilde{x}$ (I50)	70 (24)	66 (18)
**BMI (kg/m**^**2**^)*		
$\tilde{x}$ (I50)	23.0 (5.2)	23.9 (5.8)
**Handedness n (%)**		
Right	363 (93.9)	377 (92.9)
Left	25 (6.4)	29 (7.1)
**Physical activity n (%)**		
Yes	323 (83.0)	245 (60.3)
Cardio training	217 (55.8)	132 (32.5)
Strength training	55 (14.1)	48 (11.8)
Mobility training	50 (12.9)	35 (8.6)
Ball sports	54 (13.9)	14 (3.4)
Other training	189 (48.6)	121 (29.8)
**Muscle training**		
Yes	186 (47.8)	125 (30.8)

In addition to the number (n) and percentage (%), the median ($\tilde{x}$) and the interquartile distance (I50) are shown. Significant differences (Chi-square test or Wilcoxon-Mann-Whitney test) are marked with an asterisk (* at p = 0.01, ** at p = 0.02, *** at p = 0.03).

### Workplace related data of the study population

Among other self-reported things, the specializations of the Ds or of the practices in which the DAs were active were examined. With 75.8% of the Ds and 58.6% of the DAs, the participants were most frequently employed in a general dental practice. In addition, 12.1% of the Ds and 8.6% of the DAs were employed in an orthodontist practice, 4.1% of the Ds and 10.1% of the DAs in an oral surgeon/oral and maxillofacial surgeon practice and 3.1% of the Ds and 5.4% of the DAs in an endodontist practice. Other practices accounted for 4.6% of the Ds and 16.7% of the DAs, including dental hygiene/dental prophylaxis, prosthetics, pedodontology and periodontology.

Among the Ds, 49.9% were either an owner or shared owner of the practice, 30.1% worked as an employed dentist in a practice and 14.1% as an assistant dentist in a practice, while 5.7% worked as a dentist at a university hospital and 1.0% as a dentist at a general hospital.

It was determined that most Ds (46.3%) worked in a solo dental practice, while 38.0% worked in a joint practice and 10.3% in a shared practice.

Table 2 shows information on the distribution of a dental professional's working time. Significant differences (p = 0.01) were found between the working hours and the profession.

**Table 2:** Work and profession related data of the study population in n (%)–Ds and DAs.

	Ds	DAs
**Ergonomic content during studies or training**		
Yes	38 (9.8)	27 (6.7)
No	62 (15.9)	35 (8.6)
Average total working time per week in hours*		
$\tilde{x}$ (I50)	40 (10.0)	38.5 (8.0)
Not reported in n (%)	26 (6.7)	28 (6.9)
Average treatment time per week in hours*		
$\tilde{x}$ (I50)	33 (7.0)	30 (15.0)
Not reported in n (%)	26 (6.7)	35 (8.6)
Average administrative time per week in hours*		
$\tilde{x}$ (I50)	6 (6.0)	4 (7.0)
Not reported in n (%)	26 (6.7)	39 (9.6)

In addition to the number (n) and percentage (%), the median ($\tilde{x}$) and the interquartile distance (I50) are shown. Significant differences (Chi-square test or Wilcoxon-Mann-Whitney test) are marked with an asterisk (* at p = 0.01, ** at p = 0.02, *** at p = 0.03).

### MSDs prevalence

**Fig 1:**
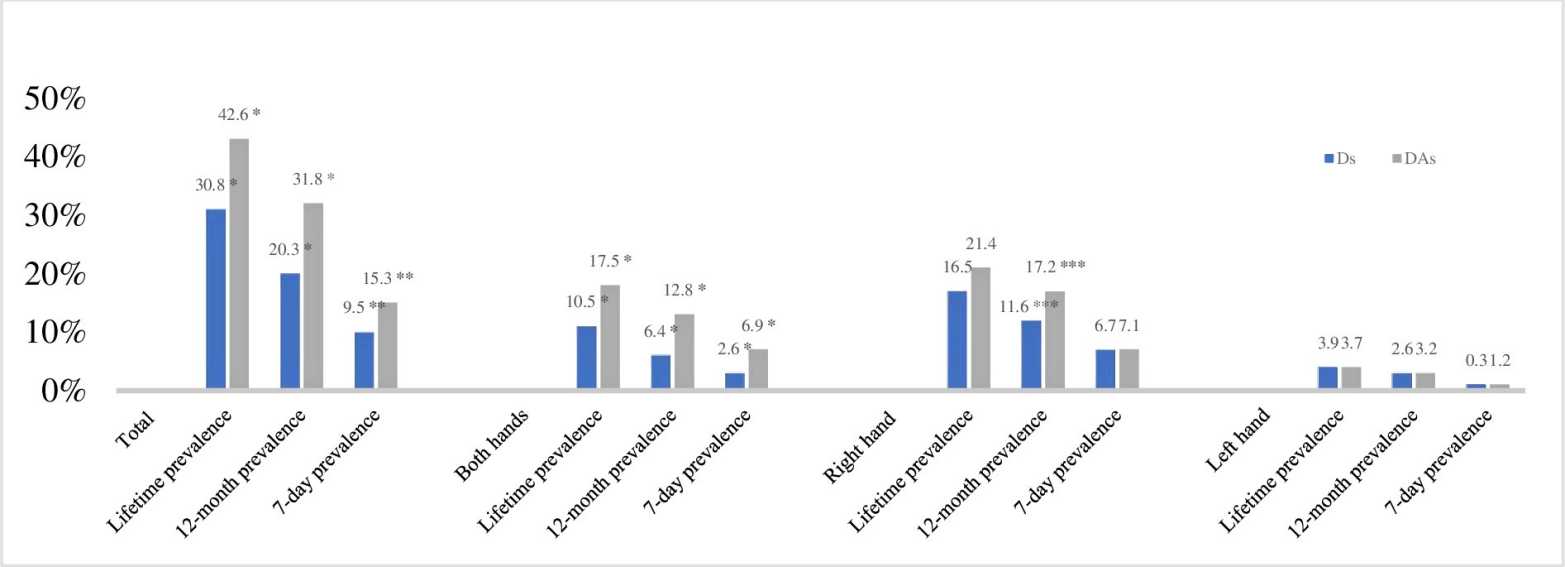
Prevalence of MSDs in the region of the hand/wrist among Ds and DAs in %. Ds are coloured in blue, DAs in grey. Significant differences (Chi-Quadrat-Test or Wilcoxon-Mann-Whitney-Test) are marked with an asterisk (* at p = 0.01, ** at p = 0.02, *** at p = 0.03).

Fig 1 describes the prevalence of the self-reported MSDs in the hand or wrist area of the Ds and DAs in Germany for comparison. The study examined lifetime, 12-months and 7-day prevalences in relation to the occurrence in total, on both hands, on the right hand or on the left hand.

In terms of overall occurrences 30.8% (n = 120) of Ds affirmed MSDs in the hand at any time in their lives, 20.3% in the last twelve months and 9.5% in the last seven days. Among the DAs, 42.6% (n = 173) reported a prevalence of MSDs in the hand at any time in their lives, 31.8% in the last 12 months and 15.3% in the last seven days.

The following significant differences were calculated between the occupational groups of Ds and DAs: total lifetime prevalence (p = 0.01), lifetime prevalence in both hands (p = 0.01), total 12-month prevalence (p = 0.01), 12-month prevalence in right hand (p = 0.03), 12-month prevalence in both hands (p = 0.01), total 7-day prevalence (p = 0.02) and 7-day prevalence in both hands (p = 0.01).

In this study female Ds were more likely to report symptoms than male Ds in all three prevalences surveyed: lifetime prevalence 37.1% and 20.8%, 12-month prevalence 23.1% and 18.0%, and 7-day prevalence 10.3% and 9.4%. The Chi-square test was used to investigate prevalence differences between female and male Ds; since 98.8% of DAs were female, only Ds were included in this test. The total lifetime prevalence of MSDs (p = 0.01) and the lifetime prevalence of MSDs in the right hand (p = 0.01) were found to be significantly different between female and male Ds.

### The prevalence of specific medical conditions

The self-reported prevalence of specific medical conditions for Ds and DAs in Germany is described in Table 3. In both the Ds and DAs occupational groups, the clinical pictures reveal arthrosis (5.1% and 6.4%, respectively) and tendovaginitis (8.0% and 11.3%, respectively) to be the most common medical conditions. However, a significant difference could only be demonstrated for rheumatism (p = 0.02).

**Table 3:** Prevalence of specific medical conditions among Ds and DAs in Germany.

	Occurrence n (%)	Occurrence before start of profession n (%)	Occurrence after start of profession n (%)	Occurrence after x years $\tilde{x}$ (I50)
Rheumatism**				
Ds	3 (0.8)	1 (0.3)	2 (0.5)	19 (0.0)
DAs	12 (3.0)	2 (0.5)	7 (1.7)	15 (20.0)
**Arthrosis**				
Ds	20 (5.1)	3 (0.8)	14 (3.6)	17 (13.0)
DAs	26 (6.4)	1 (0.2)	20. (4.9)	18 (13.0)
**Carpal tunnel syndrome**				
Ds	14 (3.6)	3 (0.8)	9 (2.3)	15 (10.0)
DAs	12 (3.0)	-	8 (2.0)	7 (16.0)
**Tendovaginitis**				
Ds	31 (8.0)	8 (2.1)	20 (5.1)	10 (14.8)
DAs	46 (11.3)	8 (2.0)	24 (5.9)	4 (7.0)
**Flexor tendovaginitis**				
Ds	3 (0.8)	-	1 (0.3)	19 (0.0)
DAs	6 (1.5)	1 (0.2)	3 (0.7)	14.5 (19.0)
**Cubital Tunnel Syndrome**				
Ds	-	-	-	-
DAs	1 (0.2)	-	1 (0.2)	-

Significant differences (Chi-Quadrat-Test or Wilcoxon-Mann-Whitney-Test) are marked with an asterisk (* at p = 0.*01*, ** at p = 0.*02*, *** at p = 0.*03)*.

For all specific medical conditions, the complaints occurred only, or predominantly, after the start of entering the dental profession.

Only for the clinical picture rheumatism a significant difference could be tested (p = 0.02).

### Therapies

**Fig 2:**
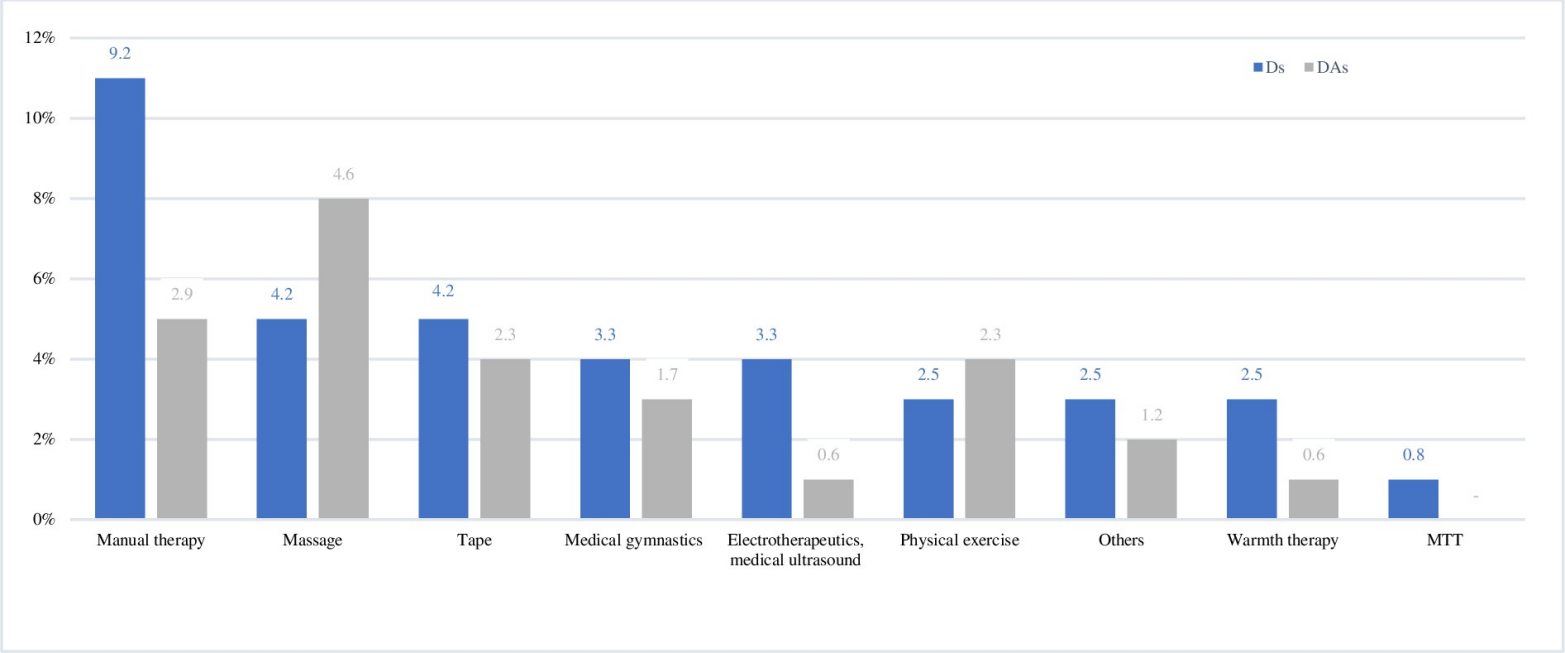
Different concepts of physiotherapy used for the therapy of hand MSDs. Ds are coloured in blue, Ds in grey. Splints and insoles are included under "Tape", TENS is included under "Electrotherapeutics and medical ultrasound" and sport is included under "Physical exercise.

Table 4 describes the self-reported use of therapy and, if used which therapy concepts have been applied by the study participants. 37.5% of the Ds and 28.3% of the DAs stated that they had been treated for MSDs. For both Ds (20.0%) and DAs (28.3%), physiotherapy was the most frequently chosen form of therapy. The selection of the different concepts of physiotherapy is described in more detail in Fig 2. 89.7% of Ds and 63.3% of DAs who received therapy reported an improvement.

**Table 4:** Therapy of hand MSDs of Ds and DAs in Germany.

	Ds n (%)	DAs n (%)
**No therapy**	75 (62.5)	124 (71.7)
**Therapy**		
** Total**	45 (37.5)	49 (28.3)
Physiotherapy	24 (20.0)	26 (15.0)
Medicine	15 (12.5)	23 (13.3)
Operation	5 (4.2)	7 (4.0)
Others***	25 (20.8)	17 (9.8)
Success of therapy (related to those who have had therapy n = 45 or 49)***		
Aggravation	1 (0.3)	1 (2.0)
No impact	2 (0.5)	17 (34.7)
Improvement	44 (11.3)	31 (63.3)
**Others**	25 (20.8)	17 (9.8)
Acupuncture	2 (1.7)	2 (1.2)
Chiropractic	2 (1.7)	0 (0.0)
Osteopathy	4 (3.3)	3 (1.7)
Ergotherapy	5 (4.2)	1 (0.6)
Physical treatments	2 (1.7)	0 (0.0)
Bandages/Tapes/Ortheses/Inlays	6 (5.0)	11 (6.4)
Physical exercise	6 (5.0)	1 (0.6)
Immobilisation	2 (1.7)	1 (0.6)

*Physical exercise included sports*, *training*, *stretching*, *Yoga*, *Pilates and relaxation*. *Physical treatments included heat*, *cold and electric applications*.

Significant differences (Chi-Quadrat-Test or Wilcoxon-Mann-Whitney-Test) are marked with an asterisk (* bei p = 0.*01*, ** bei p = 0.*02*, *** bei p = 0.*03)*.

Significant differences (p = 0.03) were found in the other therapies and in the success of therapy.

Fig 2 shows the different concepts of physiotherapy performed on Ds and DAs due to MSDs of the hand. Manual therapy was the most commonly used technique by the Ds (28%), whilst massage was more commonly used by the DAs (8%).

## Discussion

For both Ds and DAs, a prevalence of self-reported hand complaints could be described: total lifetime prevalence of 30.8% for Ds and 42.6% for DAs; a total 12-month prevalence of 20.3% for Ds and 31.8% for DAs and total 7-day prevalence of 9.5% for Ds and 15.3% for DAs. The results showed that all prevalences of the DAs were significantly higher than those of the Ds (lifetime prevalence: p = 0.01, 12-month prevalence p = 0.01, 7-day prevalence: p = 0.02).

This occupational difference may be due to the different gender distribution between the groups; 98.8% of the DAs versus 61.7% of the Ds were female (Table 1). These numbers correspond to the typical gender distribution of the respective occupational group in Germany [57]. Other studies have shown that women have an increased perception of pain and sensitivity to pain [58] and women also more readily report pain than men [59]. In the present study, a significant difference was found between the female and male Ds with the total lifetime prevalence (p = 0.01) as well as the lifetime prevalence of the right hand (p = 0.01) (the test for differences was not performed for the DAs due to the high proportion of women (98.8%)). Another potential cause for the higher prevalences of MSDs in the DAs may be due to the subordination or adjustment of DAs in their assistance work to the habits, preferences and constitutional conditions of the Ds. This observation was confirmed by several lecturers of different departments and centers for dentistry, oral and maxillofacial medicine, where the education of students of dentistry in Germany is carried out. This includes, for example, the height adjustment of the chair, the arrangement of the instruments or of the patient. Since men are, on average, taller than women (Table 1), there may be different preferred height positions in the working environment between the Ds and DAs (due to the higher proportion of women in the DAs (Table 1)). This may cause the DAs to work in an inappropriate position for them. Future ergonomic studies should investigate this possibility.

Since the occurrence of MSDs is not only caused by poor ergonomic settings, but has multifactorial influences, individual factors must also be taken into account. Physical activity is generally considered a preventive factor of MSDs [60]. Since the DAs were found to engage in significantly less physical activity than Ds (p = 0.01: Ds 83.0%, DAs 60.3%), this is, therefore, also considered to be another factor that could lead to poorer prevention of MSDs. In addition, muscle training was performed significantly less by the DAs compared to the Ds (p = 0.01: Ds 47.8%, DAs 30.8%). Besides the physical factors, the individual psychosocial environment could influence and vary the presence of MSDs among dental professionals [61]. This is a hazard that has not been analysed in the present study but which could be a starting point for further research.

Both the Ds and the DAs in the present study had a higher prevalence of MSDs in the right hand than in the left (Fig 1). This may be due to the fact that 93.9% of the Ds and 92.9% of the DAs were right-handed (Table 1). Therefore, the right hand can be assumed to be the dominant working hand.

A significant difference in the working hours (the average total working time, the average treatment time and the average administrative time) between the occupational groups was not found (Table 2) and so does not provide a plausible explanation for the different prevalences of MSDs in the Ds and DAs.

Beyond the prevalence of MSDs, the present study examined those therapies which were conducted to treat MSDs. A significant difference between the Ds and DAs was found only in "Other therapies” but, nevertheless, other trends were evident. 37.5% of the Ds suffering from MSDs stated that they had undergone therapy; physiotherapy was the most frequently mentioned type of therapy (20.0%). Although DAs have a higher prevalence of MSDs (28.3%) they reported as having had therapy less often than Ds; physiotherapy was also the most commonly reported therapy for DAs (15.0%).

Although no significant difference was found out, there is a trend that less training, in ergonomic working, or its concepts, are taught in the training of DAs than in dental studies (in the present study this was found to be 6.7% versus 9.8%). Thus, therapy concepts may be less known and less frequently conducted by DAs.

Moreover, there are two different health insurance systems in Germany. Employees (such as DAs) are usually covered by public health insurance, whereas the self-employed (such as dentists with their own practices) and employees with a high income can take out private insurance. Certain therapeutic concepts may not be covered by the statutory health insurance and so may not be affordable for DAs on their own having a lower salary compared to the Ds.

This could also be a reason why the therapies performed were significantly more successful among Ds than DAs (p = 0.01). With 63.3% of the respondents indicating an improvement through therapy, the success of the therapies was less clear for the DAs than for the Ds who enjoyed a success rate of about 99%. The reason could be that DAs have to terminate the therapy, which is not covered by health insurance, due to economic reasons. Since it is important, particularly in the case of preventive measures, to schedule the therapy sessions regularly and on a long-term basis [48], this could be a reason for the lower success rate in DAs. Furthermore, the therapies undertaken may not fully address the problems of the DAs; DAs have different strains on their hands than the Ds and, therefore, may need different preventive and therapeutic support.

Compared to many other studies [4, 8, 11–13, 18, 19], the lifetime and the 12-month prevalence of hand MSDs in Ds and DAs in the current study is lower. Advanced investigations and possibly, the implementation of ergonomic conditions in dentistry in recent years, may contribute to these differences [44]. Besides, most of the studies, including the present study, have adopted the Nordic Questionnaire [4, 11–13, 18, 19]. However, the composition of the questionnaire in this study, including additional questions not taken from the Nordic Questionnaire, differs from the composition of other studies; this may have led to the differing results. Another reason may be the heterogeneity of the dental professionals interviewed with regard to pre-existing physical conditions, specialization of the dental practice and other individual influencing factors.

It seems difficult to compare the present data with the prevalence of general musculoskeletal disorders at the hand with the general population in Germany, as there is hardly any current broad data on this topic. In a study from 1998 [62] it was investigated that between 7.0% and 21.0% of the general population in England had hand complaints. Compared to the present study, DAs and Ds have approximately from 50% up to 100% higher prevalence rates.

Some limitations of this study have been identified. The investigated group is merely a random sample of the population [63]. Furthermore, the fact that people suffering from MSDs are more interested in participating in the questionnaire than people without symptoms, can lead to a biased prevalence and to a limitation of the external validity of the study. Furthermore, questions that reach far into the past could be answered more inaccurately and unreliably. In addition, the individual statements of the complaints were not checked for a true diagnosis. Individual differences in the study population in terms of psyche and current condition [64], or in terms of body height, body weight, physical condition and diseases, may have an impact on the occurrence and perception of MSDs. Furthermore, online questionnaires have a lower response rate than paper-based questionnaires [65, 66], thus, younger people may feel more familiar with the medium of an online questionnaire than older people; indeed, this may be the reason for the younger sample (Table 1) present in this study.

Overall, the prevalence of MSDs indicates that more research should be undertaken into the mechanism of development, prevention and therapy of MSDs. This should be regularly taught and put into practice, both in training or studies and in later stages of the career [67]. The existing prevalence of MSDs in the hand area among Ds and DAs and the simultaneously relatively low therapy perception (especially among DAs), show that there is a need for future research in the field of therapies. In order to maintain or increase the success of therapies, it would be interesting to conduct further studies that analyse the success or failure of individual therapies and their contents, as well as to investigate the respondents' reasons for, or against, carrying out a therapy. For example, a therapy including medication for a complaint could be examined more closely with regard to the medication compliance. For a better understanding of the higher prevalence of MSDs in the hands of DAs compared to Ds, future research could also investigate the ergonomic risks of DAs in Germany during their work, using ergonomic risk assessment tools. Recent findings indicated that a combination of ergonomic risk assessment tools and motion capture could be a promising measure to quantify the ergonomic risk in occupational settings [68].

## Conclusion

MSDs of the hand are frequent complaints of Ds and DAs in Germany whereby the self-reported lifetime prevalence of MSDs at the hand is higher among DAs (42.6%) than among Ds (30.8%). Among the respondents with hand complaints, 37.5% of Ds and 28.3% of DAs reported having undergone therapy. The most frequently applied, self-reported therapy was physiotherapy. In summary, the prevalence of MSDs on the hand is still present in Ds and DAs, although the problem has been described for some time. At the same time, the perception of the therapies appears low compared to the prevalence numbers especially among DAs. Although the prevalence of MSDs was higher among DAs compared to Ds, both the implementation of a therapy and the success of a therapy is higher in Ds than in DAs. Consequently, there is a need for further research and attention regarding the prevalence as well as the possible therapies of MSDs at the hands.

## Supporting information

S1 Table Prevalence of MSDs in the region of the hand/wrist among Ds and DAs. (DOCX)

S2 Table Therapy of hand MSDs–physiotherapy. (DOCX)
