# Supplementary material for: Prevalence of self-reported musculoskeletal disorders of the hand and associated conducted therapy approaches among dentists and dental assistants in Germany
Source: PLoS One. 2020 Nov 6;15(11):e0241564. doi: 10.1371/journal.pone.0241564 (PMC7647093; doi:10.1371/journal.pone.0241564)
Supplement: S1 Table — (DOCX) [file pone.0241564.s001.docx]

**S1 Table*.* Prevalence of MSDs in the region of the hand/wrist among Ds and DAs.**

|  | **Ds** | **DAs** |
| --- | --- | --- |
| **Total**  **n (%)**  Lifetime prevalence  12-month prevalence  7-day prevalence | 120 (30.8*)  79 (20.3*)  37 (9.5**) | 173 (42.6*)  129 (31.8*)  62 (15.3**) |
| **Both hands**  **n (%)**  Lifetime prevalence  12-month prevalence  7-day prevalence | 41 (10.5*)  25 (6.4*)  10 (2.6*) | 71 (17.5*)  52 (12.8*)  28 (6.9*) |
| **Right hand**  **n (%)**  Lifetime prevalence  12-month prevalence  7-day prevalence | 64 (16.5)  45 (11.6***)  26 (6.7) | 87 (21.4)  70 (17.2***)  29 (7.1) |
| **Left hand**  **n (%)**  Lifetime prevalence  12-month prevalence  7-day prevalence | 15 (3.9)  10 (2.6)  1 (0.3) | 15 (3.7)  13 (3.2)  5 (1.2) |

*Ds: n 389 = 100%, DAs: n 406 = 100%, Significant differences (Chi-Quadrat-Test or Wilcoxon-Mann-Whitney-Test) are marked with an asterix (* at p = 0.01, ** at p = 0.02, *** at p = 0.03).*
