# Supplementary material for: Prevalence of self-reported musculoskeletal disorders of the hand and associated conducted therapy approaches among dentists and dental assistants in Germany
Source: PLoS One. 2020 Nov 6;15(11):e0241564. doi: 10.1371/journal.pone.0241564 (PMC7647093; doi:10.1371/journal.pone.0241564)
Supplement: S2 Table — (DOCX) [file pone.0241564.s002.docx]

**S2 Table. Therapy of hand MSDs – Physiotherapy.**

|  | **Ds (120 (100))**  **n (%)** | **DAs (173 (100))**  **n (%)** |
| --- | --- | --- |
| **Physiotherapy**  Massage (auch Schröpfen)  Manual therapy  Medical gymnastics (KG)  Physical exercise  Tape  Electrotherapeutics, medical ultrasound  Warmth therapy  MTT  Others | 24 (20.0)  5 (4.2)  11 (9.2)  4 (3.3)  3 (2.5)  5 (4.2)  4 (3.3)  3 (2.5)  1 (0.8)  3 (2.5) | 26 (15.0)  8 (4.6)  5 (2.9)  3 (1.7)  4 (2.3)  4 (2.3)  1 (0.6)  1 (0.6)  -  2 (1.2) |
